# Electrophysiological characterization of male goldfish (*Carassius auratus*) ventral preoptic area neurons receiving olfactory inputs

**DOI:** 10.3389/fnins.2014.00185

**Published:** 2014-06-30

**Authors:** Wudu E. Lado, David C. Spanswick, John E. Lewis, Vance L. Trudeau

**Affiliations:** ^1^Department of Biology, University of OttawaOttawa, ON, Canada; ^2^Department of Cell and Systems Biology, University of TorontoToronto, ON, Canada; ^3^Warwick Medical School, University of WarwickCoventry, UK; ^4^Department of Physiology, Monash UniversityClayton, VIC, Australia

**Keywords:** ventral preoptic area, male goldfish, monosynaptic glutamatergic projections

## Abstract

Chemical communication via sex pheromones is critical for successful reproduction but the underlying neural mechanisms are not well-understood. The goldfish is a tractable model because sex pheromones have been well-characterized in this species. We used male goldfish forebrain explants *in vitro* and performed whole-cell current clamp recordings from single neurons in the ventral preoptic area (vPOA) to characterize their membrane properties and synaptic inputs from the olfactory bulbs (OB). Principle component and cluster analyses based on intrinsic membrane properties of vPOA neurons (*N* = 107) revealed five (I–V) distinct cell groups. These cells displayed differences in their input resistance (R_input_: I < II < IV < III = V), time constant (TC: I = II < IV < III = V), and threshold current (I_threshold_: I > II = IV > III = V). Evidence from electrical stimulation of the OB and application of receptor antagonists suggests that vPOA neurons receive monosynaptic glutamatergic inputs via the medial olfactory tract, with connectivity varying among neuronal groups [I (24%), II (40%), III (0%), IV (34%), and V (2%)].

## Introduction

Chemical communication plays a vital role in vertebrate reproduction. Biologically-active sex pheromones have evolved across the animal kingdom to convey reproductive information to conspecifics (Dulac and Torello, 2003). However, in most cases, the neural circuitry associated with the processing of sex pheromones is poorly understood. Chemical communication is especially important in animals like goldfish because they rely on external fertilization and often live in turbid waters. These fish have evolved sex pheromones to synchronize spawning between the sexes and thus ensure reproductive success. Further, the goldfish is an attractive model to study the neural substrates of chemical communication because it is one of the few vertebrates whose sex pheromones have been fully characterized (Stacey et al., 1989; Sorensen et al., 1991; Dulka, 1993).

Studies of male goldfish indicate that sex pheromones from females regulate male sexual behavior and milt production by inducing the release of luteinizing hormone (LH) from the male pituitary gland through stimulation of gonadotropin-releasing hormone (GnRH) in the POA (Stacey, 1983; Kobayashi et al., 1986, 2002; Trudeau, 1997). The POA controls the release of LH (Peter et al., 1990; Chang et al., 2000; Trudeau et al., 2000a,b) via a signaling pathway involving dopamine (DA), which tonically inhibits both GnRH and LH release (Peter and Paulencu, 1980; Kah et al., 1987; Sloley et al., 1992; Popesku et al., 2011). Coupled to the GABAergic inputs this area receives from the ventral telencephali pars ventralis (Vv) (Martinoli et al., 1990; Trudeau et al., 2000b), the vPOA may be the site where DA suppression of GnRH is removed to allow increased GnRH levels to elicit LH release and subsequent spawning.

To characterize the neural pathways underlying the OB and POA networks, we have developed a novel *in vitro* explant preparation of the goldfish forebrain (Trudeau et al., 2000b). The adult goldfish brain is small and relatively unmyelinated making it attractive for patch clamp electrophysiology. In addition, the explant preserves the underlying neural circuitry yet allows for easy access to neurons on the ventral surface of the brain.

Here, we first describe the intrinsic membrane properties of neurons in the vPOA. Based on these properties, we suggest that vPOA neurons comprise five different subgroups. We then characterized the synaptic projections from the OB to the vPOA. In the goldfish and the closely related Crucian carp, the lateral olfactory tract (LOT) transmits food-related odors (Dulka, 1993; Hamdani et al., 2001a,b) while the medial olfactory tract (MOT) conveys exclusively pheromonal and social signals (Demski and Dulka, 1984; Sorensen et al., 1991; Hamdani et al., 2000). Here, we demonstrate that there are functional glutamatergic projections from the OB to the POA through the MOT.

## Materials and methods

### Experimental animals

This study was approved by the animal care committee of the University of Ottawa and carried out in accordance with the guidelines of Canadian Council on Animal Care. Common goldfish *(Carassius auratus)* weighing 15–40 g were purchased from a commercial supplier (Aleong's International Inc., Mississauga, ON, Canada). Fish were acclimated to 18°C, fed and maintained on a simulated photoperiod as previously reported (Trudeau et al., 1991). Only male goldfish were used throughout the study. During spawning season, sexually mature males were easily discernable by their distinctive tubercles and some readily expressed milt when their anogenital area was gently pressed. After the spawning season and during recrudescence, when tubercles are not always evident, sex was confirmed post-mortem by visual inspection of the testes.

Fish were anesthetized by immersion in 0.05% tricaine methanesulphonate (TMS) prior to dissection of the brain explant. Briefly, after severing the spinal cord, the skull was carefully opened with surgical scissors to expose the brain. The brain was dissected out from the skull cavity by cutting the optic nerves, and then removing the whole brain with olfactory bulbs still attached. The explant was attached to a Petri-dish ventral side up at the level of the spinal cord and cerebellum with cyanoacrylate glue, and placed in a bath with ice-cold artificial cerebrospinal fluid (ACSF) of the following composition [mM]: 127 NaCl, 1.9 KCl, 1.2 KH_2_PO_4_, 2.4 CaCl_2_, 1.3 MgCl_2_, 26 NaHCO_3_, 10 D-glucose; gassed with carbogen (95 O_2_, 5% CO_2)_; pH adjusted to 7.4 with NaOH. The ACSF was modified from rats' ACSF (Spanswick et al., 1998) and it was similar to others (ACSF) in other fish such as *Apteronotus leptorhynchus* (Kotecha et al., 1997) and *C. auratus* (Wilkie et al., 2008). When magnesium-free solution was used, MgCl_2_ was omitted from the ACSF.

The meninges were removed with fine forceps to expose the ventral telencephalon and access the vPOA; then a transverse cut using a razor blade was made posterior to the hypothalamus to free the brain from the dish. The brain was then transferred carefully to a custom-built recording chamber perfused at room temperature with ACSF at a rate of 2–4 ml/min. The brain explant was mounted with the ventral side up and then held between two custom-made nylon grids where it was allowed to recover for 1 h prior to recordings; all recordings were made in the 7 h following dissection. Neuroanatomical nomenclature in this study follows that of Anglade et al. (1993). Our vPOA corresponded to *nucleus preopticus periventricularis* as depicted in Plate 43 of the goldfish brain atlas (Peter and Gill, 1975).

### Electrophysiological recordings

Electrophysiological recordings were made based on previous methods for rat spinal cord and hypothalamus (Spanswick et al., 1998). Whole-cell patch clamp recordings using a Multiclamp 700B amplifier (Molecular Devices) in current clamp mode, were obtained from vPOA neurons (*N* = 107) in the *in vitro* forebrain explants at room temperature (~18–20°C) from 120 fish. Patch pipettes (5–8 MΩ) were fabricated from borosilicate filament glass (Sutter Instrument Co., Novata, CA, USA) using a horizontal pipette puller (P2000; Sutter Instrument Co., Novata, CA, USA) and filled with intracellular solution of the following composition [mM]: 140 K-gluconate, 10 KCl, 1 sucrose, 2 Na_2_ATP, 1 EGTA-Na_4_ plus 10 HEPES and pH adjusted to 7.4 with KOH modified from Spanswick et al. (1998).

Using the anterior commissure and optic chiasm as landmarks, patch electrodes were guided to the vPOA under visual control of a dissecting microscope. Seal formation was monitored on an oscilloscope. Once a gigaohm seal (typically > 5 GΩ) was achieved, whole-cell access was made by gentle suction. Series resistance was < 25 MΩ.

To measure synaptic connectivity, a bipolar stimulating electrode was inserted into one of the olfactory bulbs (OBs). Postsynaptic potentials (PSPs) in the vPOA were elicited by single pulse electrical stimulation (5–30 V, 0.2 ms pulse duration) of the ipsilateral OB via a stimulus isolation unit (Digitimer Ltd., model DS2). Data acquisition and experimental control was performed using pCLAMP 9.2 software (Molecular Devices). Data were low-pass filtered at 2 kHz and acquired at 10 kHz and later analyzed offline using CLAMPFIT 9.2 software (Molecular Devices).

### Pharmacological agents

To characterize the pharmacological properties of the connectivity from the OB to the vPOA neurons, we used 6-cyano-7-nitroquinoxaline-2, 3-dione (CNQX; Tocris), an α-amino-3-hydroxy-5-methyl-4-isoxazolepropionic acid (AMPA/kainate) receptor antagonist; and D-2-amino-5-phosphonopentanoic acid (D-APV; Tocris), an N-Methyl-D-aspartic acid or N-Methyl-D-aspartate (NMDA) receptor antagonist. Drugs were made up as stock solutions, CNQX in DMSO (Sigma-Aldrich) and D-APV in distilled water, then, diluted in Mg^2+^ free ACSF. The final concentration of DMSO was always < 0.1%. Typically, Mg^2+^ free ACSF and the drugs were applied sequentially for 10 min each to the recording chamber before any attempts at recordings to allow sufficient equilibration time.

### Data analysis

**Figure 1:**
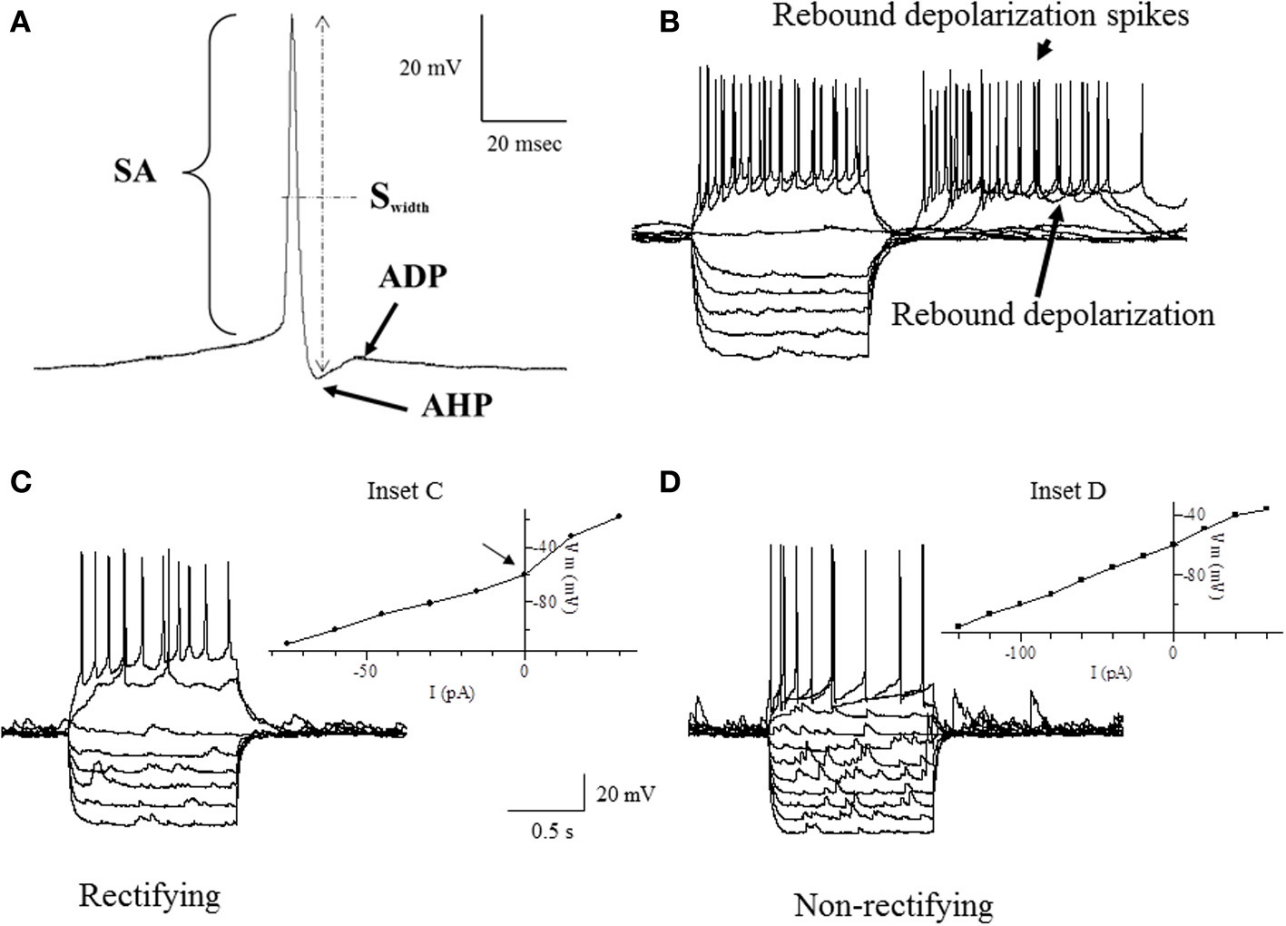
**Traces of some of the intrinsic membrane properties used in our statistical analyses. (A)** Depicts the AHP (after hyperpolarization potential), APA (action potential amplitude), APD (action potential duration) and ADP (after depolarization potential). **(B)** Shows the RD (rebound depolarization) and RDS (rebound depolarization spikes). **(C,D)** demonstrate the current to voltage relationship (IV) where **(C)** is rectifying as shown by the arrow at the inflection point (Inset C) and **(D)** is non-rectifying (Inset D). APA was measured from the shoulder at threshold shown by the arrowhead. APD was the width of the AP measured from the shoulder at threshold shown by the arrowhead. See definitions in Materials and Methods section.

Intrinsic membrane properties of vPOA neurons were characterized by patch clamp electrophysiology to determine whether they constituted distinct populations. After achieving whole cell access, the resting membrane potential (RMP) which is the baseline potential in the absence of any current stimulus was measured in current clamp mode (*I* = 0 nA). In addition, properties related to spontaneous action potential production was measured (Figure 1A): Spike amplitude (SA) was measured from the shoulder of the rising phase (~threshold) to the peak; spike width (S_width_) was measured at the width of half-maximal from the peak to the afterhyperpolarization (AHP); AHP was determined from the threshold to the peak of the hyperpolarization following the action potential (note that only the fast component of the AHP was considered in this study); after-depolarization potential (ADP) was measured from the hyperpolarization peak to the ADP peak. For neurons that showed spontaneous activity, the coefficient of variation of the interspike interval (the interval between successive spikes) was calculated over a 90 s time window (CV_spikes_ = SD/mean). Neurons were then stimulated with 1 s hyperpolarizing and depolarizing current steps (2–30 pA) from a holding potential of −60 mV to measure a number of other intrinsic membrane properties (Figures 1B–D): current threshold (I_threshold_) is the minimum current required to elicit an action potential; spike threshold (S_threshold_) is the number of spikes elicited by the minimum current i.e., at I_threshold_; the presence or absence of a rebound depolarization (RD) after a hyperpolarizing current step; the presence or absence of rebound depolarization spikes (RDS), rebound spike frequency (RS) is the action potential frequency during the RD; the presence or absence of H current (I_H_) which is associated with non-selective cation channels; rectification (Rec) refers to a non-linearity in the current-voltage (IV) relationship (Siegelbaum and Koester, 2000) (compare insets in Figures 1C,D); the input resistance (R_input_), which is the slope of the IV curve; membrane time constant (TC) is the time for the hyperpolarization response to reach two-thirds of its plateau value; soma membrane capacitance (C_soma_) is calculated as TC/ R_input_ (Abbud and Smith, 1995).

A principle component analysis (PCA; SPSS Inc.; 2006, v.15) was used to reduce the set of intrinsic membrane properties to a number of independent uncorrelated variables. For this analysis, all properties that were characterized by their presence or absence, i.e., RD, RDS, I_H_ and Rec were assigned binary values (0, 1). The PCA variables were then used to cluster (SPSS Inc.; 2006, v.15) the neurons into groups. An unsupervised cluster analysis was performed to classify neurons, similar to previous studies (Ward, 1963; Krimer et al., 2005; Sosulina et al., 2006; Andjelic et al., 2009). This method consisted of grouping individual neurons based on the Euclidean distance between their respective PCA loadings.

**Figure 2:**
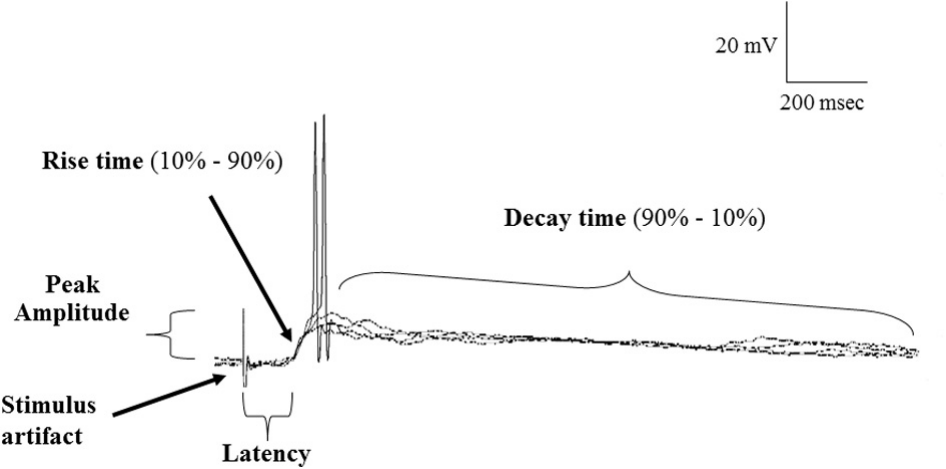
**Samples of a continuous recording showing superimposed EPSPs evoked in POA neurons following stimulation of the olfactory bulb under normal (ACSF) conditions and highlighting the properties of EPSPs measured**. Note that evoked EPSPs could give rise to action potential firing and showed constant latency and rise time, consistent with a monosynaptic origin.

Postsynaptic potentials (PSP) data were characterized by the peak amplitude (the height of the evoked PSP measured from baseline to peak), the latency (the time between the OB stimulus and beginning of PSP rise), 10–90% rise time (measured from the shoulder of the rise to the peak) and 90%–10% decay time (determined from 90% of the peak of the PSP to 10% above baseline) (Figure 2). To measure variability in the latency and rise time, the mean, standard deviation (SD) and coefficient of variation (CV); (SD/mean) was calculated over 4 stimulus trials in each cell.

All data generated by the PCA were tested for normality and homogeneity of variance, and either an analysis of variance (ANOVA) or a Kruskal-Wallis (KW) analysis was used for between group comparisons where appropriate (SPSS Inc.; 2006, v.15). *Post-hoc* analyses consisted of paired *t*-tests and Tukey's (SPSS Inc.; 2006, v.15). Unless otherwise stated, data are reported as mean ± s.e.m.

## Results

### Heterogeneity of intrinsic membrane properties

To characterize the population of vPOA neurons, a number of intrinsic membrane properties were quantified (see Materials and Methods). We used a Principle Component Analysis (PCA) to determine the set of properties that could best distinguish neuronal subgroups. The PCA revealed seven significant properties (loading factor) [RD (0.92), R_input_ (0.87), SA (0.77), S_threshold_ (0.80), RS (0.82), I_H_ (0.78), and Rec (0.83)] (Table 1). A subsequent cluster analysis of these variables revealed five distinct neuronal subgroups (denoted I, II, III, IV, and V). In the following, we compare the membrane properties across the different subgroups (Table 2).

**Table 1:** **The rotated component matrix of variables used in our principle component analysis**.

**Property/Loaded factors**	**1**	**2**	**3**	**4**	**5**	**6**	**7**
RMP (mV)	−0.247	0.151	−0.075	−0.014	−0.217	0.692	−0.314
TC (msec)	0.224	0.769	0.210	−0.065	0.120	−0.160	0.228
R_input_ (GΩ)	−0.113	**0.870**	0.030	0.026	−0.151	0.041	−0.035
AHP (mV)	−0.320	0.152	0.664	−0.081	0.000	−0.085	−0.240
SA (mV)	0.209	0.003	**0.770**	−0.104	0.033	−0.261	0.159
I_threshold_ (pA)	−0.056	−0.368	0.057	0.794	−0.001	−0.238	0.120
S_threshold_ (Hz)	0.228	0.299	0.037	**0.797**	−0.057	0.122	−0.073
S_width_ (ms)	−0.061	−0.043	−0.784	−0.340	0.043	−0.189	−0.092
CV	−0.038	0.285	−0.172	0.218	−0.720	−0.086	−0.225
RS	0.171	0.086	−0.079	0.056	**0.782**	−0.018	−0.126
ADP (mV)	0.397	−0.397	0.223	−0.173	−0.409	−0.051	−0.148
I_H_	0.041	−0.184	−0.058	−0.053	−0.094	**0.783**	0.305
Rec	−0.134	0.135	0.031	0.031	0.039	0.044	**0.819**
RD	**0.916**	−0.066	−0.002	0.040	0.076	−0.093	0.032
RDS	0.877	0.136	0.007	0.136	0.135	−0.035	−0.179

Loaded factors in bold show measurements that were subsequently used for our cluster analysis. See definitions in Materials and Methods section and Figure 1. Extraction Method: Principal Component Analysis. Rotation Method: Varimax with Kaiser Normalization. Rotation converged in 8 iterations.

**Table 2:** **Intrinsic membrane properties of POA neurons measured in ACSF**.

**Property/Clusters**	**I (*N*** = **23)**	**II (*N*** = **38)**	**III (*N*** = **4)**	**IV (*N*** = **36)**	**V (*N*** = **6)**
RMP (mV)	−56.5 ± 2.2	−58.2 ± 1.4	−57.1 ± 4.5	−56 ± 1.4	−54.3 ± 2.1
TC (ms)	47.6 ± 5.2	47.0 ± 2.7	92.9 ± 15.3	66.2 ± 3.9	110.3 ± 10.8
R_input_ (GΩ)	0.42 ± 0.04	1.0 ± 0.03	3.7 ± 0.15	2.0 ± 0.07	5.9 ± 0.04
C_soma_ (pF)	144.3 ± 24	45.8 ± 2.8	24.9 ± 3.7	34.3 ± 3.4	19.2 ± 2.8
AHP (mV)	12.2 ± 1.3	10.2 ± 0.8	13.7 ± 5.8	11.0 ± 1.0	15.4 ± 3.7
SA (mV)	56.1 ± 2.7	52.6 ± 2.3	60.7 ± 5.7	53.1 ± 1.9	54.3 ± 4.5
I_threshold_ (pA)	13.0 ± 2.0	8.2 ± 1.2	3.9 ± 0.8	6.0 ± 0.7	3.3 ± 0.8
S_threshold_ (Hz)	3.3 ± 0.6	2.4 ± 0.3	2 ± 0.4	3.5 ± 0.4	5.3 ± 1.0
CV	1.1 ± 0.1	1.1 ± 0.1	1.0 ± 0.1	1.1 ± 0.1	2.3 ± 0.6
ADP (mV)	1.4 ± 0.4	1.6 ± 0.4	–	0.6 ± 0.2	0.12 ± 0.0

*RMP (resting membrane potential); TC (membrane time constant); R*_input_ *(input resistance); C_m_ (membrane capacitance); AHP (after hyperpolarization potential); APA (action potential amplitude); TI (action potential threshold current); ST (number of spikes at threshold current); APD (action potential duration); CV (coefficient of variation); ADP (after depolarization potential). Not shown are variables for rectifying and RS. See definitions in Materials and Methods section and Figure 1.*

**Figure 3:**
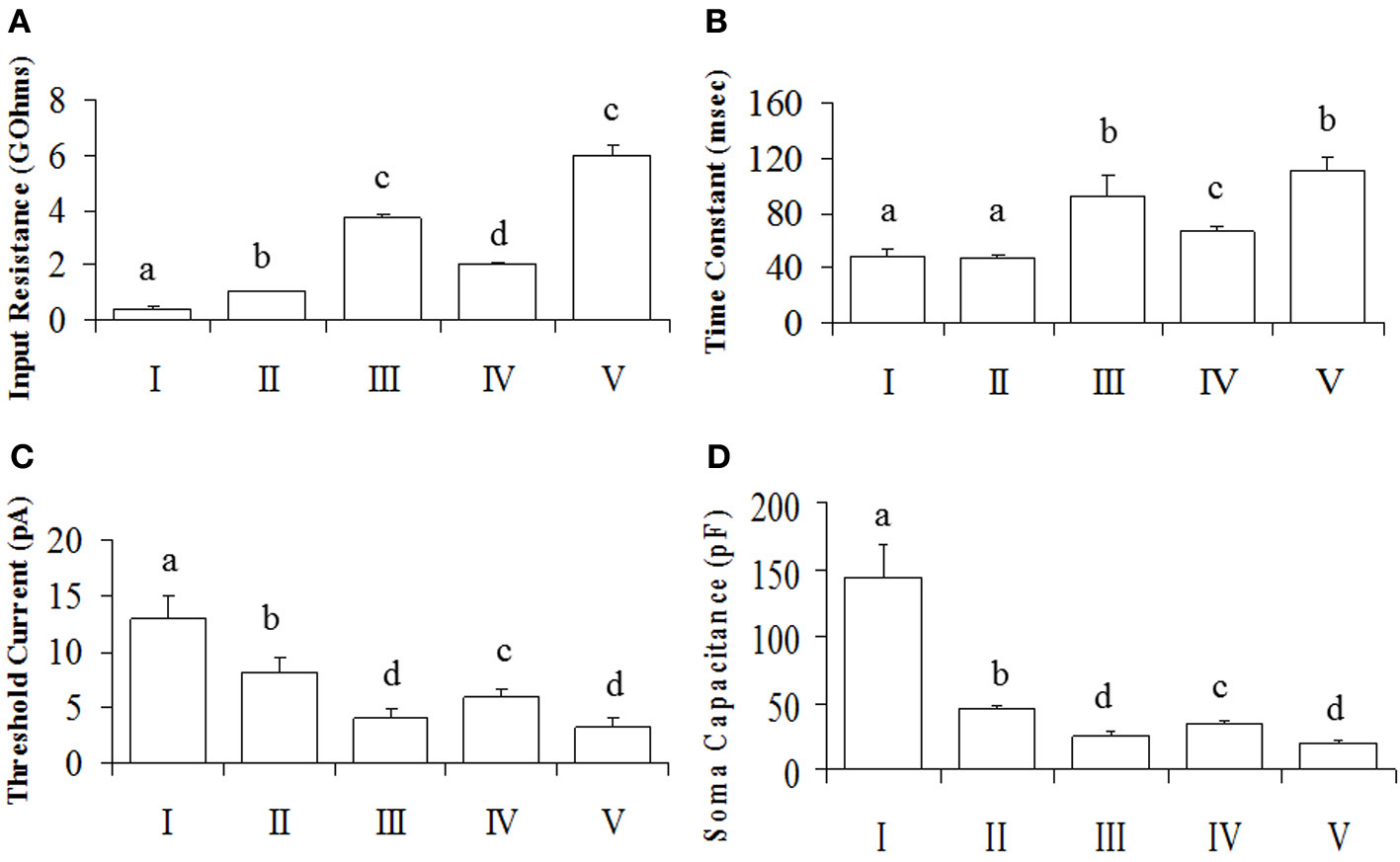
**Intrinsic membrane properties of POA neurons**. Neurons were made up of connected (*N* = 50) and unconnected (*N* = 57) neurons to the OB. **(A)** The input resistance showing group differences. **(B)** The time constant, indicating differences between groups. **(C)** The threshold current differences between groups. Results are presented as mean ± s.e.m. for convenience. Letters represent groups that differed significantly (*P* < 0.005) from each other with Bonferroni corrections. **(D)** Soma capacitances of the different neuronal groups.

Since the data from our PCA failed the normality test (*P* > 0.05), it was transformed to its square root equivalent; and statistical analyses performed. Neuronal subgroups were found to differ in their R_input_ [*F*_(4, 106)_ = 325.93, *P* = 0.001], TC [*F*_(4, 106)_ = 13.63, *P* = 0.001] and I_threshold_ [*F*_(4, 106)_ = 3.86, *P* = 0.006] but not ADP [*F*_(4, 106)_ = 1.35, *P* = 0.25]. *Tukey's post-hoc* analyses showed that R_input_ was different in each neuronal cluster with V = III > IV > II > I (Figure 3A). Similarly, the TC of neurons in clusters III and V were higher than IV which in turn was higher than those in clusters I and II (Figure 3B). In addition, I_threshold_ for neurons in cluster I was greater than II which in turn was greater than for IV greater than III and V neurons (Figure 3C). Since the rectification (Rec) and RS were categorical variables, *post-hoc* comparisons were performed using KW analyses: rectification was not significant [χ^2^_(4)_ = 1.55; *P* = 0.818] nor was RS [χ^2^_(4)_ = 10.28; *P* = 0.036, Bonferroni correction, *P* > 0.005].

Overall, our analyses showed that only R_input_, TC and I_threshold_ were significantly different between groups (*P* < 0.05), while rectification and RS were not (*P* > 0.05). Together, the R_input_, TC and C_soma_ constitute the passive membrane properties of the cell. Note that the TC is directly proportional to the product of the R_input_ and C_soma_ [TC = R_input_. C_soma_] (Molleman, 2003). The calculated C_soma_ (after its transformation to the reciprocal of its square root to normalize the data) was also statistically different between neuronal groups [*F*_(4, 106)_ = 25.89, *P* = 0.001], with *Tukey's post-hoc* indicating that I > II > IV > III = V (Figure 3D). Since capacitance is proportional to membrane area (Hille, 2001), the differences we observe between neuronal groups can be at least partially explained by neuronal size.

### Properties of postsynaptic potentials: inputs from the olfactory bulb

Given these putative subgroups of vPOA neurons, we next set out to determine their inputs from the olfactory bulb (OB). Of the 107 vPOA cells tested, 50 received synaptic inputs from the OB. The ratio of connected to unconnected neurons in each cluster was: I: 59% (12/23); II: 60% (20/38); III: 0% (0/4); IV: 53% (17/36); and V: 19% (1/6).

The PSPs were reliably evoked with latencies of approximately 90 ms. The small coefficients of variation (CV) of the latency (1.5%) and rise-time (2.2%) within cells are not consistent with a multi-synaptic pathway (for which latency is expected to be more variable). Given the consistency of the synaptic response, we suggest that these connections are monosynaptic (Spanswick et al., 1998). Since the distance from the OB to the POA is about 9 mm, a latency of 100 ms suggests an estimated conduction velocity similar to the slowest conduction velocity (~0.1 m/s) reported in olfactory nerves of the tench at similar temperatures (Dubois-Dauphin et al., 1980).

### Biochemical properties of the postsynaptic potentials of vPOA

**Figure 4:**
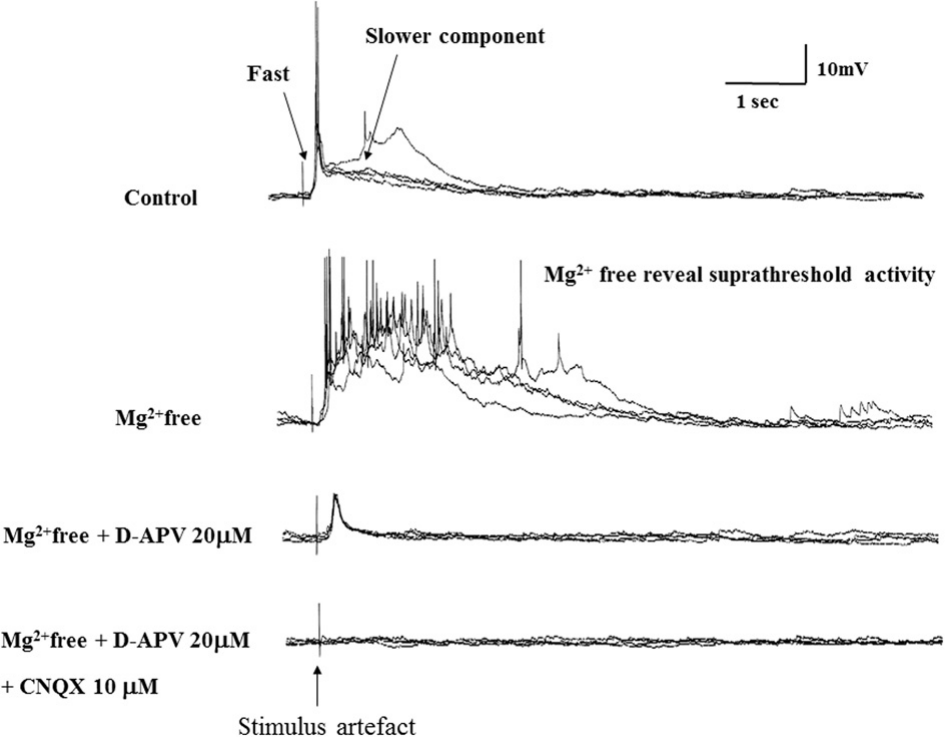
**Evoked EPSPs in a POA neuron under different recording conditions**. After achieving whole cell access, recordings were made in current-clamp mode. Typically, neurons were perfused sequentially with normal ACSF, Mg^2+^free ACSF, 20 μM AP-5 (in Mg^2+^free ACSF) and 10 μM CNQX (in Mg^2+^free ACSF) for 10 min each before recordings.

To characterize the pharmacological properties of the PSP in vPOA (*N* = 13) neurons, goldfish brain explants were perfused sequentially with normal ACSF, Mg^++^-free ACSF (MFACSF), 20 μM D-APV and 10 μM CNQX before washing off both drugs with normal saline. The latency, peak amplitude rise and decay times of the evoked PSPs were then measured and compared under the different recording conditions. Figure 4 shows representative data from a vPOA neuron. The Mg^2+^free ACSF increased the PSP compared to normal saline. The glutamate antagonist APV partially blocked the evoked response. The residual response was subsequently blocked completely by CNQX, indicating that the PSPs had a dual component and were mediated by glutamate acting on both NMDARs and AMPARs (Figure 4). Indeed, when AMPARs alone were blocked with CNQX (data not shown; *N* = 10), no EPSPs were evoked suggesting that activation of NMDARs requires preceding depolarization via AMPARs to overcome voltage-dependent Mg^2+^ block.

**Figure 5:**
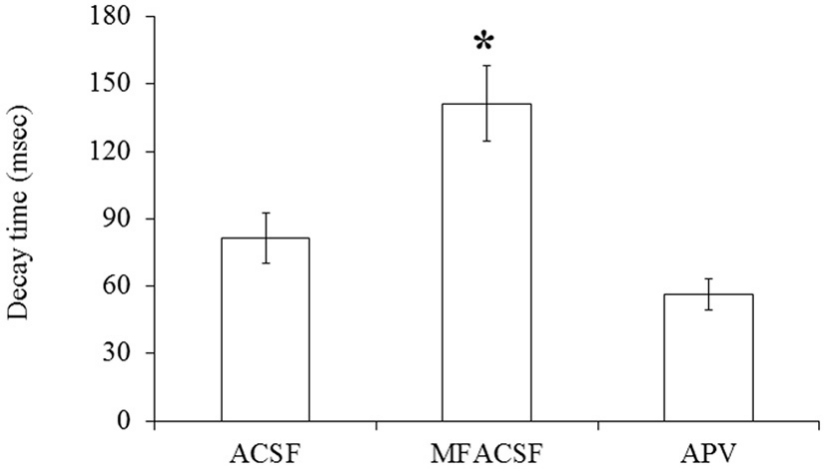
**The PSP decay time of POA neurons connected to the OB under the different perfusions (normal ACSF, Mg^2+^ free ACSF (MFACSF) and APV)**. Error bars denote s.e.m. Asterisks (^*^) statistical significance (*P* < 0.05) between perfusions.

A statistical analysis on data obtained from POA neurons (*N* = 13) did not find significant differences (*P* > 0.05) in their PSP latencies and rise times between the different perfusion media, but their decay times differed significantly between MFACSF vs. ACSF [*F*_(1, 12)_ = 16.11, *P* = 0.002] and MFACSF vs. APV [*F*_(1, 12)_ = 16.59, *P* = 0.002]. *Post-hoc* analysis indicated that POA neurons in MFACSF had a longer decay time than in either ACSF [*t*_(13)_ = 3.76, *p* = 0.002] or APV [*t*_(13)_ = 4.07, *p* = 0.002] (Figure 5). These data show that the evoked PSPs had a biphasic response, with APV partially blocking the slower and longer lasting NMDAR component, revealing a faster and shorter lasting AMPAR component that was completely blocked by CNQX.

### Sectioning the lateral olfactory tract while recording from the vPOA

To determine if the OB to vPOA projection is mediated through the MOT, we sectioned the LOT (*N* = 3) while leaving the medial tract intact. We found that PSPs evoked in vPOA neurons by OB stimulation did not differ from those evoked under control conditions for latency, rise time, peak amplitude and decay times (KW analysis; *P* > 0.05). Cutting the MOT (*N* = 10) while leaving the LOT intact did not elicit any response consistent with studies which have shown that the MOT innervates targets in area dorsalis and area ventralis while the LOT projects only to area dorsalis (Levine and Dethier, 1985). Furthermore, pharmacological manipulations influenced the recorded EPSPs in similar ways for both cut and intact LOTs for latency, rise time, peak amplitude and decay times (KW analysis; *P* > 0.05). Sectioning both the medial and LOTs abolished the PSPs completely. This confirms that the pathway we describe from OB to POA is via the MOT.

## Discussion

Female sex pheromones regulate reproduction in male goldfish through the olfactory system (Partridge et al., 1976; Sorensen et al., 1991; Dulka, 1993; Stacey et al., 2003; Chung-Davidson et al., 2008). Previous anatomical studies have shown direct neuronal pathways from the OB to POA in the teleost brain (Forlano and Bass, 2011). With whole-cell patch clamp recordings, we show for the first time that these connections are functional and glutamatergic. In addition, we show that these synapses involve both N-methyl-D-aspartate (NMDAR) and α-amino-3-hydroxy-5-methyl-4-isoxazolepropionic acid receptors (AMPAR). We also provide evidence, with a thorough characterization of electrophysiological properties, that neurons of the vPOA comprise several subgroups.

The POA is an important hypophysiotropic center that regulates reproduction in vertebrates. Electrical stimulation of this area has been shown to elicit sperm release (Demski, 1983; Dulka and Demski, 1986; Dulka, 1993; Dominguez, 2009); sexual calling (Schmidt, 1968); nest-building and courtship (Demski and Knigge, 1971) in several vertebrate models. Conversely, lesioning the POA impairs reproduction in male goldfish thereby underscoring the importance of this neural system (Hart et al., 1973; Kyle and Peter, 1982; Kyle et al., 1982; Koyama et al., 1984; Sorensen et al., 1991; Dulka, 1993).

### Heterogeneity of neuronal electrical properties

We recorded from neurons in the vPOA while stimulating the OB. Our analysis revealed five subgroups of vPOA neurons, each with distinct intrinsic membrane properties and variable connectivity to the OB. Indeed, the POA has been shown to contain a plethora of cells immunoreactive to substance P (Sharma et al., 1989), GnRH (Peter et al., 1990, 2003; Parhar et al., 2001), γ-aminobutyric acid (GABA) (Martinoli et al., 1990), glutamate (Anglade et al., 1993), somatostatin (Canosa et al., 2004), CRF (Olivereau et al., 1984), secretoneurin (Canosa et al., 2011) vasotocin (Parhar et al., 2001) and tyrosine hydroxylase (Hornby et al., 1987). Thus, this heterogeneity in electrophysiological profiles may reflect functionally diverse classes of vPOA neurons. Further work is required to determine if these electrophysiological “signatures” correspond to chemical phenotypes, exhibiting differential projections and functional roles.

### Properties of postsynaptic potentials: inputs from olfactory bulb

Electrical stimulation of the OB evoked PSPs in vPOA neurons. These PSPs gave rise to action potentials at the peak of their responses in some cases. The evoked excitatory PSPs (EPSPs) had consistent and constant latencies and rise times with small coefficient of variation suggesting they arise through monosynaptic inputs from the OB (Spanswick et al., 1998). The conduction velocities of the inputs from the OB to the POA were similar to those found previously in other systems (Gasser, 1956; Potapov and Gusel'nikova, 1976b). The relatively slow conduction is consistent with propagation through unmyelinated olfactory fibers (Westerman and Wilson, 1968; Potapov and Gusel'nikova, 1976a; Farbman, 1992). The conduction velocity of the inputs from the OB to the vPOA was estimated to be 0.1 m/s, which was similar to the pike (Gasser, 1956) and slower, by four times, than that reported by Kandel (1964) in the goldfish for the larger POA magnocellular neurons projecting to the neural lobe of the pituitary gland when stimulated antidromically.

### Biochemical properties of the synaptic connections

Anatomical connections between the OB and vPOA have been established previously through tract-tracings (Levine and Dethier, 1985; Anglade et al., 1993), but their functional nature remains unclear. To investigate the possible role of glutamate in chemical communication, we perfused the goldfish brain explant sequentially with ACSF, APV, and CNQX in Mg^2+^free ACSF while stimulating the OB to measure the latency, amplitude, rise time, decay time and duration of the evoked potentials in the vPOA (Figure 4). Mg^2+^free ACSF enhanced the evoked EPSPs compared to normal ACSF. The NMDAR antagonist APV partially blocked the EPSPs leaving a fast acting and short lasting component that was subsequently completely blocked by CNQX, suggesting that the evoked PSP was mediated by glutamate acting on NMDARs and AMPARs, respectively. Receptors for AMPA may therefore be required to depolarize the cells (from their resting state) sufficiently to relieve the Mg^2+^ blockage of NMDARs (Gotz et al., 1997; Spanswick et al., 1998). Bath application of drugs allows for the possibility that the observed effects are indirect and involve peripheral pathways. However, given that the latency and rising phase of the evoked response is very consistent and remains so during drug application, the effects are likely direct.

The complete blockage of the evoked PSPs by the glutamatergic antagonists suggests that glutamate plays an important role in mediating chemical communication between the OB and vPOA. Since the POA is important for the regulation of reproductive behaviors, it may receive pheromonal cues from the OB to integrate milt release and spawning in male goldfish (Kyle and Peter, 1982; Kyle et al., 1982). The use of glutamate signaling through NMDA receptors may therefore be a mechanism to induce the sustained neuronal firing required to trigger an LH surge when sex pheromones are detected. To our knowledge, this is the first pharmacological characterization of second order neurons in the teleost olfactory system linked to reproduction.

A monosynaptic glutamatergic connection from the OB to the vPOA complements and extends our understanding of the neural circuitry involved in the control of goldfish reproduction. Previously, Trudeau et al. (2000b) demonstrated the existence of monosynaptic GABAergic projections from the Vv to the vPOA. Indeed, GABA plays a central role in male goldfish reproduction by suppressing the DAergic inhibition of LH release (Trudeau et al., 1993). This suggests that there are interactions between diverse sets of neurotransmitters and neurohormones that regulate reproduction in male goldfish. The Vv may therefore modulate the glutamatergic inputs from the OB to the vPOA to regulate some aspects of reproductive behavior or hormone release.

### Role of glutamate in goldfish reproduction

Previous studies have shown that intraperitoneal injections of male goldfish with either monosodium glutamate (MSG) (Sloley et al., 1992) or NMDA (Trudeau et al., 2000b) or AMPA (Trudeau et al., 2000b; Popesku et al., 2011) rapidly induces LH release. Furthermore, in rainbow trout it has been shown that the LH response to NMDA is blocked by APV or a GnRH receptor antagonist, indicating that glutamate modulates LH release through stimulation of GnRH (Flett et al., 1994), similar to the situation in mammalian models (Kocsis et al., 2003). Moreover, Peter et al. (1980) has shown that MSG injections in goldfish causes cellular degeneration in the POA, demonstrating excitotoxic actions of glutamate on POA neurons. Additionally, in rats it has been shown that glutamate injections in the POA or electrical stimulation of the POA decreases the latency between intromissions thereby increasing ejaculation frequency (Dominguez, 2009). Glutamate in the POA therefore plays an important role in vertebrate reproduction.

### Sectioning the lateral olfactory tract

We employed olfactory tract sectioning to determine if the glutamatergic projection to the vPOA was via the MOT or the LOT. The EPSPs recorded in vPOA cells in explants with a transected LOT had the same amplitude and duration as those with the LOT intact. These EPSPs were modulated by APV and CNQX in the same way as in intact preparations. In other experiments, sectioning the MOT while leaving the LOT intact did not elicit PSPs in vPOA neurons indicating that the OB to vPOA projection we have studied is via the MOT and not the LOT. This supports previous studies indicating unequivocally that sex pheromones signals in goldfish are mediated exclusively by the MOT indeed tract tracing studies have shown that while the MOT projects to area dorsalis and area ventralis of the telencephalon the LOT only innervates targets in the area dorsalis (Levine and Dethier, 1985; Sorensen et al., 1991).

## Conclusion

We describe an electrophysiological basis for classifying neurons of the vPOA. Further, we provide evidence that the synaptic connections from the OB to the vPOA are monosynaptic and glutamatergic. These connections from the OB to vPOA may play a role in facilitating spermiation and steroidogenesis (Peter and Paulencu, 1980; Peter et al., 1980; Kyle and Peter, 1982). While speculative at this point, the olfactory glutamatergic projections we identified may represent pathways that integrate pheromonal signals from females that stimulate reproductive hormone release and male sexual behavior in the spawning period.

## Author contributions

Wudu E. Lado, Vance L. Trudeau and David C. Spanswick designed the experiment. Wudu E. Lado and David C. Spanswick performed the experiment. Wudu E. Lado, John E. Lewis and Vance L. Trudeau analyzed the results and wrote the paper.

### Conflict of interest statement

The authors declare that the research was conducted in the absence of any commercial or financial relationships that could be construed as a potential conflict of interest.
